# The availability and functionality of medical equipment and the barriers to their use at comprehensive specialized hospitals in the Amhara region, Ethiopia

**DOI:** 10.3389/frhs.2024.1470234

**Published:** 2025-01-07

**Authors:** Alem Endeshaw Woldeyohanins, Nigatu Mihretu Molla, Abibo Wondie Mekonen, Abrham Wondimu

**Affiliations:** ^1^ Department of Social and Administrative Pharmacy, School of Pharmacy, College of Medicine and Health Science, University of Gondar, Gondar, Ethiopia; ^2^ Department of Immunization Supply Chain Management, Pharmaceutical Supply Service Head Quarter and UNICEF Ethiopia Country Office, COVID-19 and Routine Immunization Vaccine TA-Manger, Addis Ababa, Ethiopia; ^3^ Department of Veterinary Pharmacy, Pharmaceutical Supply Chain Management, University of Gondar, Gondar, Ethiopia

**Keywords:** medical equipment, availability, functionality, comprehensive specialized hospitals, Amhara region, Ethiopia

## Abstract

**Background:**

Public healthcare practices, particularly disease prevention, screening, diagnosis, treatment, and rehabilitation of patients, heavily rely on the availability and functionality of medical equipment. The absence of sufficient medical equipment and the malfunctioning of existing equipment impede the ability to provide effective healthcare services and directly affect patient rehabilitation, while the challenges related to medical equipment utilization are huge, especially in countries with limited resources such as Ethiopia. Therefore, this study aimed to assess the availability, functionality, and barriers associated with the use of medical equipment at public comprehensive specialized hospitals in Amhara Regional State, Ethiopia.

**Methods:**

A cross-sectional study design involving both quantitative and qualitative methods was conducted. Eight (*n* = 8) *comprehensive* specialized hospitals in the Amhara region were selected purposefully and included in this study. The data used to assess the availability and functionality of medical equipment items (*n* = 78) listed by the Ethiopian Ministry of Health that are supposed to be available in all of the *comprehensive* specialized hospitals were collected from 29 May to 18 June 2023. Self-administered structured questionnaires, observational checklists, and key informant interview guides were used to collect the necessary data. To analyze the quantitative data, descriptive statistics were employed, and qualitative data were analyzed using a thematic approach.

**Results:**

The study revealed that the availability of medical equipment in at least one hospital was 55.93% on average, and the availability of at least one piece of medical equipment in the surveyed hospitals was only 25.6%. The overall functional status of medical equipment was 74.68%. The present study also indicated that 75% of the surveyed facility's biomedical engineers did not receive on-the-job training regularly. Of the eight surveyed facilities, only one had spare parts and accessories for their medical equipment and the majority (87.5%) of the facilities did not have enough medical equipment storage space and did not have medical equipment policies. The qualitative findings of this study showed that issues with the utilization of the Medical Equipment Management Information System, a lack of spare parts and accessories, the absence of a well-equipped and standardized maintenance workshop, and insufficient operator training were the major challenges.

**Conclusion:**

This study revealed critical deficiencies in medical equipment availability, functionality, and barriers to maintenance at the surveyed facilities. Therefore, to improve healthcare service delivery, collaborative efforts and targeted interventions are essential in optimizing the availability and functionality of medical equipment at each and every health facility.

## Background

As recognized by the World Health Organization (WHO), health is a fundamental right for all individuals. A responsive health system ensures that communities have fair access to necessary medical equipment that is guaranteed to be safe, effective, and of high quality. The continuous advancements in science, medical equipment, and technology used for healthcare services should be of high quality and used appropriately to address global health challenges. Medical equipment is a crucial instrument for health interventions and is utilized in illness prevention, diagnosis, treatment, and patient rehabilitation (1, 2). The utilization of medical equipment involves standardization, maintenance, restoration, operator training, and retirement. However, in low- and middle-income nations, it might be difficult to obtain functional medical equipment (3, 4).

According to estimates from the WHO, in underdeveloped nations and even in Europe, Asia, and Central America, between 50% and 80% of medical equipment is broken, non-functional, and scarce, which makes it difficult for the healthcare system to provide patients with care, and the issue is highly prevalent, particularly at hospitals (HLs) in resource-limited countries (2, 5). A study conducted in South Africa showed that medical equipment availability and functionality and inadequate maintenance facilities were the main problems in the hospitals and healthcare services (2). For the successful provision of healthcare services, hospital environments should provide access to and proper application of medical equipment because patient examination and scientific diagnosis are heavily reliant on the availability of functional medical devices (1, 6). A study conducted in the United Republic of Tanzania indicated that every level of government was working to allocate more human and financial resources. For medical device management and to ensure the correct functioning of medical devices, calibration, training, maintenance, and repair workshop centers at the national, regional, and district levels were established (6).

The introduction of an electronic medical device management system provided health facilities and the Health Ministry with data on the operational status of medical devices and the need for repairs and spare parts. In developing countries, the lack of planned preventive maintenance, insufficient maintenance capacity, limited knowledge on proper usage, scarcity of accessories and spare parts, and inadequate budget for repairs contribute significantly to the non-functionality of medical equipment and hinder its proper utilization (5). Ethiopia adopted the Ethiopian Hospital Service Transformation Guidelines (EHSTG) which has played a critical role in emphasizing the importance of managing medical equipment. In particular, chapter 15 of the Hospital Services Transformation Guidelines (HSTG) describes the steps that hospitals should take to manage their medical equipment properly, enabling them to expand their services while maintaining patient safety. However, the provision of quality medical healthcare services delivered by Ethiopian health facilities has been hampered by improper management of medical equipment (5).

Data from the Medical Equipment Information Service for healthcare facilities under the Amhara Regional Health Bureau (ARHB) reveals that among the reported 8,260 pieces of medical equipment, 837 (10.1%) were non-functional and several were not installed. Furthermore, random supervisory visits to hospitals in eight zones indicated that of the 1,200 pieces of medical equipment observed, 368 were non-functional and 64 were not installed. In addition, a study conducted at public hospitals in Bahirdar and supervisory visits to health centers indicated similar issues and widespread challenges in the region (7, 8).

Access and on-time maintenance of medical equipment at each and every public health facility are essential for the provision of successful healthcare services; the lack and non-functionality of medical equipment pose a critical challenge for the delivery of health services and can lead to patients’ lives being endangered. Hence, evaluation of the availability and functionality of medical equipment and contributing factors associated with medical equipment utilization practices at a service delivery point, particularly in comprehensive specialized hospitals (CSHs), is essential to know the status of the facilities and to provide future insight for policymakers and future researchers to address the existing issues and design effective strategies. In Ethiopia, studies conducted at public primary hospitals and health centers indicated the presence of different barriers related to the accessibility and functionality of medical equipment. Furthermore, reports also showed medical equipment availability and functionality were the major challenges to providing the services and are sources of patient dissatisfaction (8–11). However, little is known about the status of medical equipment utilization practices and the reasons behind the inadequate accessibility and functionality of medical equipment in health facilities, and there are no studies that have been conducted at CSHs in the current study area, i.e., Amhara regional state, Ethiopia. Therefore, the objectives of this study were to assess the availability, functionality, and utilization practices of medical equipment and explore the potential challenges in the management of medical equipment in eight (*n* = 8) CSHs within the Amhara region, Ethiopia.

## Methods and materials

### Study area and period

The ARHB has a responsibility to promote both preventive and curative health services in the region. In the region, there are 98 hospitals (8 CSHs, 15 general hospitals, and 75 primary hospitals). Furthermore, the Amhara region has 917 health centers, 3,725 health posts, 5 health science colleges, 5 public health institutions (4 branches), 10 blood banks, and 2 oxygen plants. However, the current study was conducted in all (*n* = 8) the comprehensive specialized public health hospitals in Amhara national regional state, namely, the Felege Hiwot, Tibebe Ghion, Gondar University, Debre Markos, Debre Birhan, Dessie, Debre Tabor, and Woldiya comprehensive specialized hospitals, from 29 May to 18 June 2023.

### Study design

A cross-sectional study design involving both quantitative and qualitative methods was conducted in all the public comprehensive specialized hospitals found in the Amhara region. The quantitative data used to assess the availability and utilization status of medical equipment were collected from the selected institutions using self-administered questionnaires and observational checklists, and to explore the associated challenges, qualitative data were collected using interview guides.

### Study population

To collect the necessary data, the capital medical equipment available in all eight CSHs found in the Amhara region of Ethiopia was considered. For the qualitative data, the biomedical engineers, the pharmacy heads, the heads of the biomedical engineering section at ARHB, and a representative of Ethiopian Pharmaceutical Supply Services (EPSS) were interviewed.

### Sample size determination

Due to the limited number of comprehensive specialized hospitals found in the study area, all eight (*n* = 8) CSHs found in the Amhara region, namely Gondar University CSH (GUCSH), Tibebe Ghion CSH (TGCSH), Debre Markos CSH (DMCSH), Felegehiwot CSH (FCSH), Debre Birhan CSH (DBCSH), Dessie CSH (DCSH), Wolideya CSH (WCSH), and Debre Tabor CSH (DTCSH), were purposefully included. Regarding medical equipment, all medical equipment items included in an unpublished list from the Ministry of Health (MOH) and ARHB were assessed during the survey. Concerning the key informants (KIs), all the heads of the biomedical engineering and pharmacy departments of each hospital and the biomedical engineers at ARHB were interviewed.

### Data collection tools and procedures

Before collecting the actual data used for this study, ethical approval was obtained from the research ethics review committee of the College of Medicine and Health Sciences, School of Pharmacy at the University of Gondar with reference number (Ref No: S/A/P/41/2015). A formal letter was then written to each of the eight CSHs from ARHB. Furthermore, an informed consent form then was prepared for participants and this was provided to the respondents to get their consent. Once their consent was given, the prepared questionnaires were distributed to each participant, and then the quantitative data were collected using a self-administered questionnaire and observational checklists, whereas the qualitative data were collected using face-to-face interviews. Throughout the study, the confidentiality and anonymity of the participants were maintained. To assess the availability and functional status of a list of medical equipment, a structured questionnaire, an observational checklist, and a KI interview guide were developed by reviewing different related literature and customized as a local context to our study (4, 8–10, 12, 13). Two biomedical technicians participated in collecting the data after 1 day of training was provided to them about the objectives of the study and data collection methods. The quantitative data were collected from the heads of the biomedical engineering departments in each hospital. A self-administered questionnaire was used to collect sociodemographic data for the study facilities and participants, an observational checklist was used to collect data on the availability and functional status of the medical equipment, and the interview guide with some probing questions was used to collect qualitative data. The qualitative data were collected by the principal investigator using the KI interview guide (Supplementary Table S1). The selection of KIs was based on their knowledge of and direct involvement in medical equipment management. Accordingly, a total of 16 KIs (hospital biomedical heads, pharmacy heads, and heads of biomedical engineers) at the selected facilities were interviewed. Sound recording was employed during the interviews to ensure accurate documentation of the information provided. In addition, field notes were made during the interview.

### Data quality assurance

To ensure the quality and consistency of the data, training was given to the data collectors and supervisors, focusing on the objectives of the study, the nature of medical equipment management, and data collection tools and procedures. The investigator and supervisor closely supervised the data collection system and the data collectors. The supervisor and investigator checked the data for completeness on a daily basis during and after collection.

### Data entry and analysis

Before data entry, the collected data was checked for completeness and consistency and was then categorized and coded. The coded data was entered into MS Excel 2013 and SPSS version 26. Descriptive statistics were carried out for the quantitative data, and the results were summarized using tables, charts, and graphs. For qualitative data, the recorded audio in Amharic obtained from the KIs and the field notes made during the interview were coded, transcribed, and translated into English. Furthermore, for better identification of each theme, the interviewer repeatedly listened to the audio record and read the written notes. Finally, the data were analyzed manually using a thematic analysis. The qualitative results were then used to support the quantitative results with particular themes.

## Results

### Sociodemographic profile of health facilities

For this study, a total of eight CSHs were surveyed with a 100% response rate, and all the selected participants participated in filling out the questionnaire. Accordingly, eight participants participated, and all participants were male. The majority (7, 87.5%) had a first degree in pharmacy and one was a druggist.

### Assessment of availability of medical equipment

Of the 78 medical equipment items expected to be available in hospitals, only 20 (25.6%) (at least one) were found to be available in all the specialized hospitals (Table 1). Medical equipment such as a phaco-vitrectomy machine that is used to break up and remove cataract lenses, fluorescence microscopes, a blood gas analyzer, 300 L/500 L/700 L plasma freezers, a 64-slice computed tomography (CT) scan machine, an endoscope, a digital x-ray dental colposcope, and the like were available only at 50% (*n* = 4) of the surveyed CSHs, while the majority (*n* = 7, 87.5%) of the surveyed CSHs did not have medical equipment such as that required for optical coherence tomography (OCT), a urine analyzer, and a stress testing machine (Table 2). Five of the pieces of medical equipment were not available in any of the hospitals, namely, a lithotripter, which is used in urinary stone treatment, a sterilizer (chemical H_2_O_2_), a low-temperature freezer (500 L/700 L), an external beam radiation therapy machine (teletherapy), and the equipment required for electrotherapy modalities [throat, ear and nose (TENS), ultrasound, and combination therapy].

**Table 1:** List of medical equipment available at least one in all CSHs in Amhara region in June 2023.

List of medical equipment available in all CSHs
CPAP (continuous positive airway pressure)
ECG machine
Oxygen concentrator
Clinical chemistry fully automated analyzer
Mechanical ventilator: ICU
Monitor: patient
Monitor-fetal/maternal, electronic fetal monitoring (EFM)
Pulse oximetry
Electrohydraulic operating table
Electrosurgical unit
Operating light: ceiling
Operating light: mobile
Slit lamp (a microscope with a bright light for eye examination)
Analyzer: hematology, five differentials
Incubator: newborn
Anesthesia machine
Blood bank: refrigerated
X-ray: radiography, Digital
Ultrasound: Ob/Gyn
Ultrasound: general purpose, color Doppler, mobile, 3D

CSHs, comprehensive specialized hospitals; ECG, echocardiograph; ICU, intensive care unit; Ob/Gyn, obstetrics/gynecology.

**Table 2:** List of medical equipment available in four or fewer CSHs in the Amhara region in June 2023.

Name of medical equipment	Medical equipment available in HLs (N)
Central patient monitor	2
Steam sterilizer, 500 L	3
Ophthalmic cryo unit	2
Ophthalmic ultrasound	3
Phaco-vitrectomy machine (used to break up and remove cataract lenses)	4
Fundus camera	2
OCT	1
Perimetry/automated visual field analyzer	3
Argon laser for glaucoma, Rx	1
Ophthalmic laser (YAG/argon/SLT)	3
Urine analyzer	1
Blood gas analyzer	4
Inverted microscope	1
Microscope with video camera	1
Fully automated ELISA analyzer	3
Fluorescence microscope	4
Fully automated rotary microtome	2
Auto slide stainer—cytology/histology	2
Plasma freezer, 300 L/500 L/700 L	4
Inspissator—TB culture preparation	2
Rotary microtome, Manual	3
1.5 T MRI machine	2
64-slice CT scan machine	4
Dialysis machine	3
Digital C-arm fluoroscopy x-ray machine,	3
Digital with fluoroscopy x-ray—radiography	3
Digital x-ray—Mammography	2
Digital X-ray—dental	4
Treadmill	1
Stress testing machine	1
Ultrasound—general purpose, color Doppler, mobile, 4D	4
Endoscope—colposcope	4
Endoscope—hysteroscope	2
Endoscope—video gastrointestinal	2
Endoscope—video bronchoscope	3
Endoscope—video laryngoscope	2
Endo-urology set	2

HL, hospital; SLT, selective laser trabeculoplasty.

**Figure 1:**
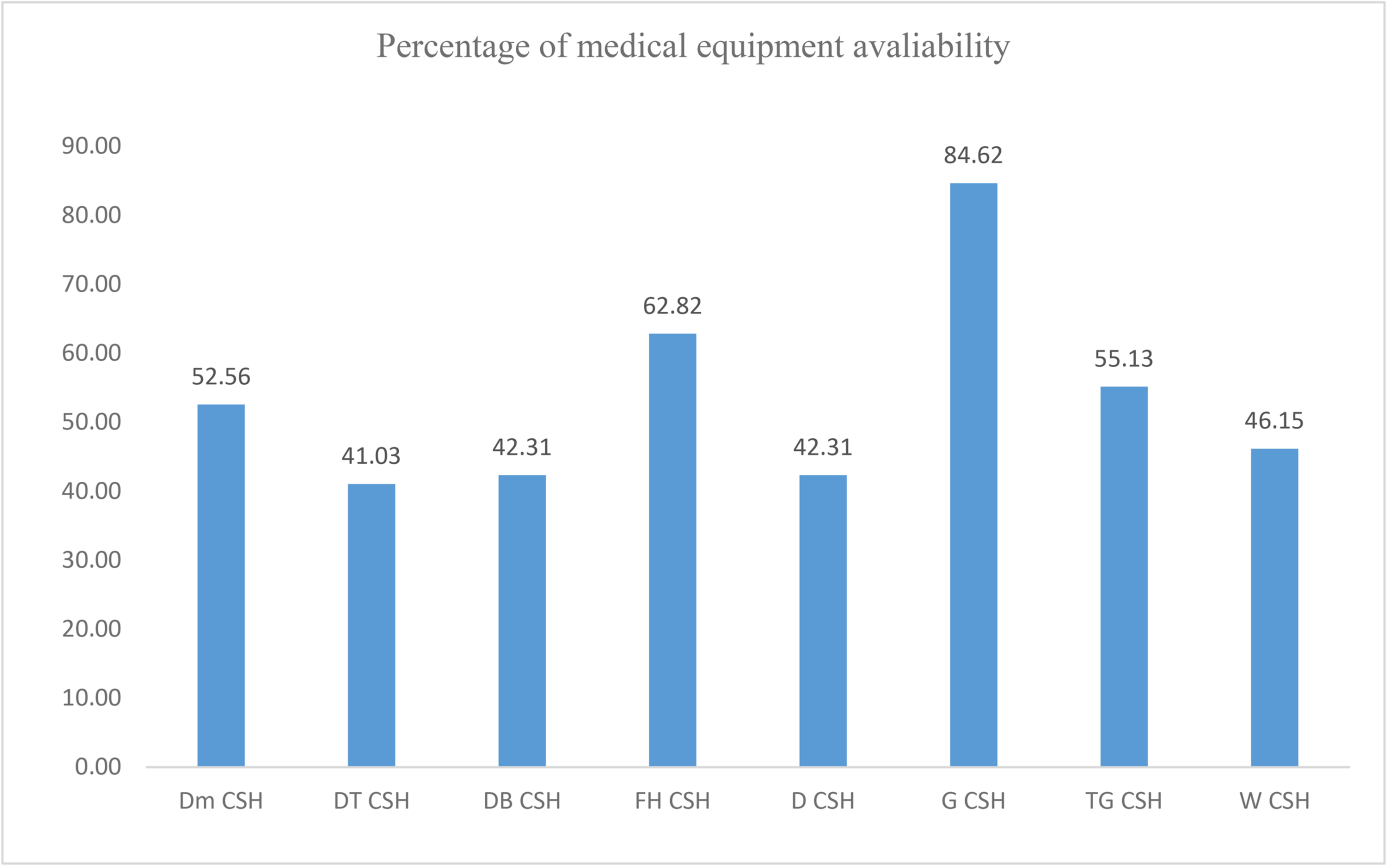
Availability of medical equipment by facility type. DMCSH, Debre Markos Comprehensive Specialized Hospital; DTCSH, Debre Tabor Comprehensive Specialized Hospital; DBCSH, Debre Berhan Comprehensive Specialized Hospital; FHCSH, Felege Hiwot Comprehensive Specialized Hospital; DCSH, Dessie Comprehensive Specialized Hospital; GCSH, Gondar Comprehensive Specialized Hospital; TGCSH, Tibebe Ghion Comprehensive Specialized Hospital; WCSH, Wollo Comprehensive Specialized Hospital.

Moreover, the availability of medical equipment in CSHs was only 55.93% on average. This study indicates that the percentage availability of medical equipment in the surveyed CSHs in the Amhara region varied from facility to facility. The highest availability was recorded at Gondar CSH (84.65%) and the lowest was at Debre Tabor CSH (41.03%) (Figure 1).

### Assessment of the functional status of medical equipment

**Figure 2:**
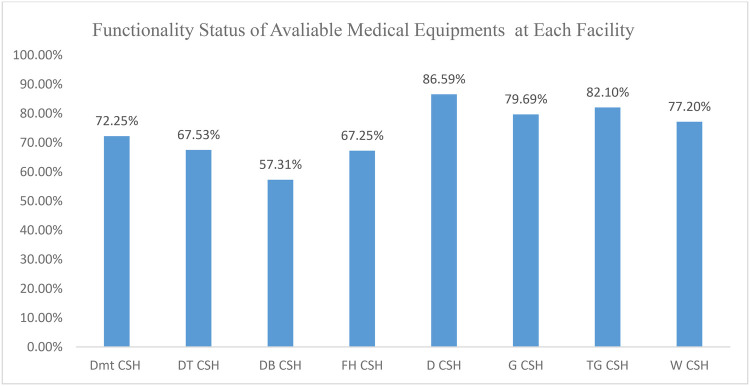
Distribution of the functional status of medical equipment in CSHs in the Amhara region in June 2023. DMCSH, Debre Markos Comprehensive Specialized Hospital; DTCSH, Debre Tabor Comprehensive Specialized Hospital; DBCSH, Debre Cerhan Comprehensive Specialized Hospital; FHCSH, Felege Hiwot Comprehensive Specialized Hospital; DCSH, Dessie Comprehensive Specialized Hospital; GCSH, Gondar Comprehensive Specialized Hospital; TGCSH, Tibebe Ghion Comprehensive Specialized Hospital; WCSH, Wollo Comprehensive Specialized Hospital.

**Figure 3:**
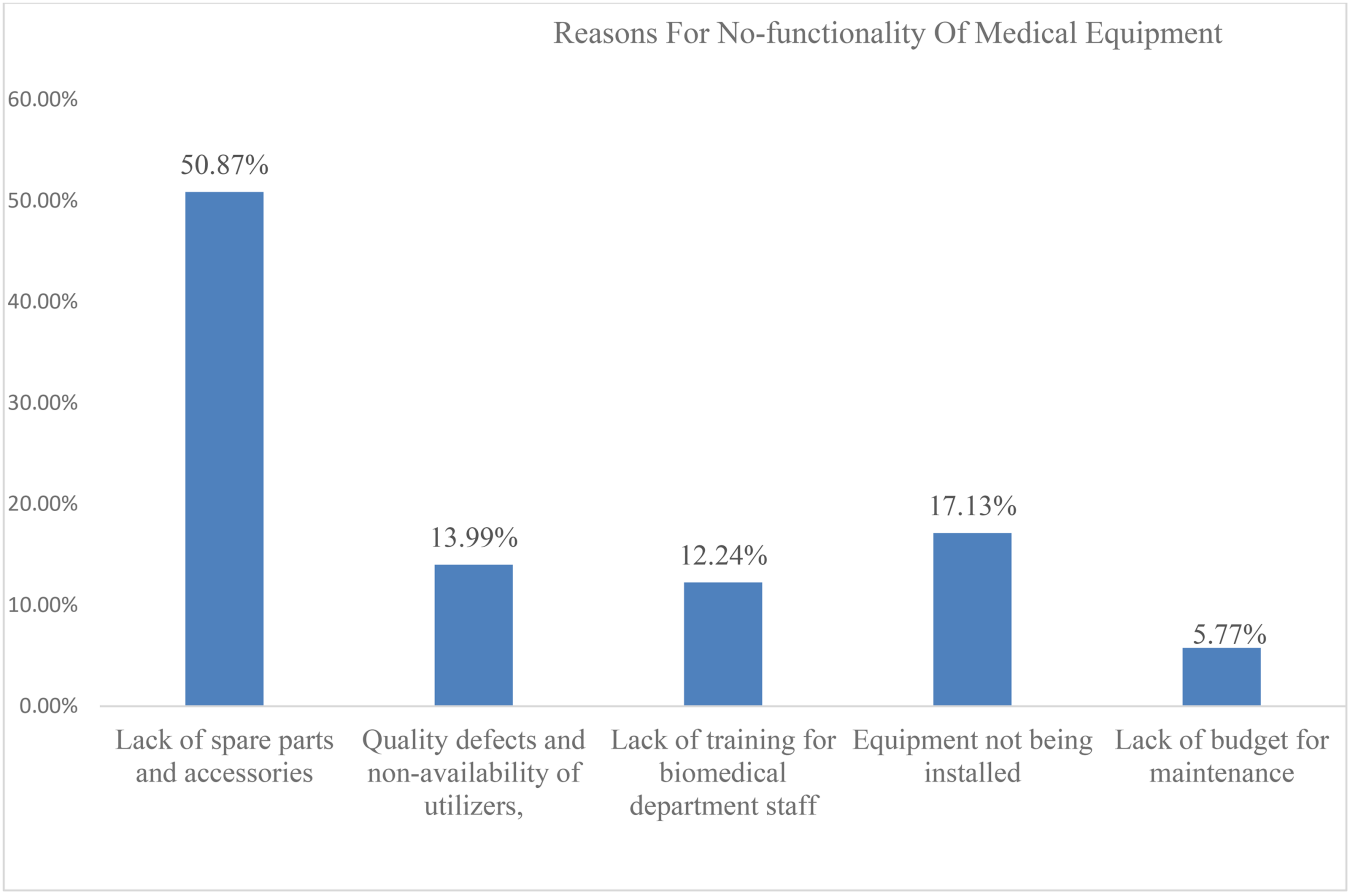
Reasons for the non-functionality of medical equipment in percent at CSHs of Amhara region (2023).

This study revealed that the total number of available pieces of medical equipment at the surveyed facilities was 2,259; among them, 1,687 (74.7%) were functional and the remaining 572 (25.32%) were non-functional (Figure 3; Table 3). The functionality status varied from hospital to hospital and ranged from 57.3% in DBCSH to 86.6% in DCSH (Figure 2).

**Table 3:** Distribution of the availability and overall functional status of medical equipment in CSHs in the Amhara region in June 2023.

Name of HL	Available ME (N)	Non-functional ME (N)	Non-functionality (%)
DMCSH	227	63	27.75
DTCSH	148	48	32.43
DBCSH	260	111	42.69
FHCSH	345	113	32.75
DCSH	276	37	13.41
GCSH	458	93	20.31
TGCSH	352	63	17.90
WCSH	193	44	22.80
Total	2,259	572	25.32

HL, hospital; ME (N), number of pieces of medical equipment; DMCSH, Debre Markos comprehensive specialized hospital.

This study also assessed the reasons behind the non-functionality of equipment available at the CSHs. Accordingly, the most common reasons for the non-functionality of medical equipment were the lack of spare parts and accessories, which accounted for 50.87%, followed by the difficulty of installing the equipment (17.13%), and other reasons for the non-functionality of medical equipment, as shown in Figure 3.

The study revealed that professional, medical equipment, and health facility-related aspects were the major issues. Regarding professional matters, the regularity of refresher training for the biomedical engineers varied; only two confirmed that they received refresher training regularly with the arrival of new medical equipment (Supplementary Table S2). Concerning medical equipment-related aspects, only two of the eight respondents confirmed the immediate installation of all medical equipment upon delivery. The majority of the respondents, seven of eight, confirmed that the hospitals do not provide spare parts before medical equipment stops working and they do not have the required accessories for the equipment. There were mixed responses to a question regarding the practicality of medical equipment preventive maintenance based on a schedule or on demand, with two “yes” and six “no” responses (Supplementary Table S3).

Regarding the health facility-related aspects, most of the hospitals (six out of eight) had a comprehensive medical equipment policy covering preventive maintenance and usage; however, only one effectively implemented the policy. Only four of the eight hospitals surveyed for this study reported the availability of operating manuals for medical equipment. A preventive maintenance schedule, based on the nature of the equipment, was in place in six hospitals. Only a few (three out of eight) hospitals continuously monitored and evaluated the use of medical equipment. Storage space for medical equipment was believed to be sufficient and appropriate only in one hospital. From the surveyed hospitals, only half (4 out of eight hospitals) had sufficient supply of power to operate all medical equipment had mixed responses, with four “yes” and four “no” responses (Supplementary Table S4).

### Qualitative data results

A total of 16 heads of the biomedical and pharmacy departments in each CSH and ARHB biomedical engineers were interviewed. All the respondents were male, and most of the respondents had more than 5 years of service experience in their current profession. The results obtained from the qualitative study were summarized under three themes, namely, the challenges related to the source of medical equipment, the installation procedures and spare parts, and the maintenance and follow-up practices of the medical equipment.

### Theme 1: the source of the medical equipment

The results obtained from the KIs revealed that the source of medical equipment was through purchasing, personal gifts, or donations (breakdown from the Ministry of Health and ARHB through EPSS).

Most of the KIs from a hospital highlighted that “medical equipment is obtained by a push system from EPSS, RHB, and MOH, directly from the project, or determined by high-level management” (Informant #1 and #4) (the facilities did not determine the quantity of equipment needed). Another KI highlighted that “Even the source of some medical equipment is not known; the donated and personal gift medical equipment's are not need-based and do not consider the skill and capability of hospitals” (Informant #7).

### Theme 2: challenges and barriers related to the installation of, and spare parts for, medical equipment

After the medical equipment is procured, it is installed immediately either by the hospitals or the company based on the agreement, and user training is provided once the medical equipment is installed. However, the qualitative results of this study indicated that if the medical equipment is obtained through a donation or gift, it takes longer to install the equipment, and there is a delay in the installation of the procured medical equipment. As identified by the KIs, a lack of spare parts and accessories is also a major barrier to the proper utilization and functionality of medical equipment in the CSHs in the Amhara regional state.

The KIs highlighted that: “Most of the donated laboratory medical equipment was closed, and it is difficult to install and to have preventive maintenance since the medical equipment is delivered without a necessary spare part”. The KIs emphasized this by saying that “if the equipment is obtained through donation, the equipment has been stored without installation for a long period of time, sometimes it couldn't be installed by the hospital biomedical engineer, and the process could be outsourced to the manufacturing company or other firms”. “This leads to extra costs and causes problems with customer service satisfaction” (Informant #1, #4, #7 and #12).

Another KI stated: “In our hospital, there is no planned medical equipment purchasing system, and even we didn't participate in the specification, purchasing, and receiving processes. Even though most of the donated laboratory medical equipment was closed and delivered without a necessary spare part, it was difficult to install and to have preventive maintenance” (Informant #7).

The majority of the KIs highlighted that: “As you know, according to HSTG Chapter 15, donated medical equipment should be delivered with a two-year supply of accessories and spare parts, along with a one-year warranty period; however, most donated medical equipment is not provided according to donation guidelines, and without an operation and maintenance manual, it lacks spare parts and accessories. Even if it is delivered without a warranty period” (Informants #4 and #16). “To be honest, most donor institutions considered Ethiopia a disposal site for medical equipment because a lot of old medical equipment was delivered through donations with incomplete accessories, rendering them unable to provide the intended service. Consequently, the medical equipment is forced to be stored for extended periods, occupying space due to being uninstalled because of the absence of spare parts and accessories. In our hospital, there is much nonfunctional medical equipment, which is due to the absence of spare parts and accessories” (Informant #7).

One KI emphasizes that “in our hospital, we have a list of 800 pieces of medical equipment but are unable to obtain spare parts and accessories. The hospital can only procure spare parts for approximately 20 pieces of medical equipment per year. Most management units were committed to allocating budget for medical equipment's spare parts, tool kits, and accessory procurement, but the spare parts were not available in the local market” (Informant #2). Another KI also added that, “even though the hospital allocates budget to purchase spare parts, it is difficult to get them in the local market. This is due to the presence of many brands for single ME (e.g., microscope has more than 20 brands), which hinders the availability of specific accessories and spare parts. It is difficult to make purchases for all due to the limited number of suppliers and the non-availability of spare parts” (Informant #3).

### Theme 3: the maintenance and follow-up practices of the medical equipment

The majority of the KIs reported that their hospital did not implement planned preventive maintenance as recommended by the manufacturer. Most key informants revealed that in most of the hospitals, there were medical equipment management protocols, and chapter 15 of the EHSTG clearly provided for and mentioned medical equipment management in hospitals. However, the information obtained from KIs indicated that the protocol is not fully implemented.

The majority of the KIs highlighted that “there is no medical equipment decommission guideline in the hospitals or even in the ministry of health. There were no written documents to manage the hospital medical equipment” (Informants #4, #7, #9, and #16). The other critical challenge mentioned was a lack of commitment to use the Medical Equipment Management Information System (MEMIS). Most of the KIs mentioned that: “In the MEMIS, there are 15 key performance indicators, and among them only the hospitals use the MEMIS for inventory purposes. However, if the MEMIS performed as intended, most challenges would be solved. This is because the MEMIS software was interoperable with medical equipment suppliers, medical equipment manufacturers, EPSS, MOH, and all administrative units of the MOH, including HFs. There were no regular follow-ups from the management, and there was no monitoring and evaluation system in place, and hospitals and RHB were not enforced to implement MEMIS for medical equipment management and were not evaluated as per the Key Performance Indicators (KPIs)” (Informants #7, #9, and #16).

The KIs highlighted the need to improve the donation practice and system by saying that “Rather than donating several medical equipment items with high value but old and outdated technology, it is better if donors donate a smaller number of new and up-to-date medical equipment that have spare parts and accessories in the local market, and the medical equipment donation practices must have a monitoring and evaluation system; therefore, decision makers should be informed about the current situation of the medical equipment and different models and types of medical equipment that help a lot to maintain and repair accordingly” *(*Informants #4, #9, #12, and #16).

The absence of well-equipped, well-designed, and well-organized medical equipment maintenance workshops with basic units (such as mechanical, testing, administrative, electrical, biomedical, and software) and skilled biomedical engineering professionals were also mentioned as critical challenges.

One KI highlighted that the majority of the hospitals did not provide regular workshops. “As far as I know, there is no national medical equipment maintenance workshop to serve as a training center” (Informant #16). This KI added that “Even if there is a preventive maintenance schedule, however, the preventive maintenance is not performed as per the schedule in most of the departments, and curative maintenance is not conducted in our hospitals except for selected medical equipment at emergency, ICU, and OR departments. The biomedical engineers are not well trained with the available medical equipment; the work order is not filled by the users, and it is not reported timely there for how we are efficient in practicing curative maintenance.” (Informant #16)

Most of the KIs emphasized that “the hospital management and Ministry of Health didn't prioritize preventive maintenance, resulting in non-functional medical equipment worth millions due to minor and less expensive accessories or spare parts.” (Informants #9, #12, and #15)

Closed-system medical equipment, power interruptions, a lack of tailored training for equipment users/operators and biomedical engineers, and a lack of support from the management unit were other challenges frequently raised by the majority of key informants. The KIs mentioned that “when the closed system medical equipment failed, it was costly to maintain the equipment since placement agreement is only with the manufacturer or the agent. It is difficult to provide regular service with the available medical equipment due to a power interruption.” (Informants #2, # 4, #6, and #10)

Most of the KIs indicated that the curriculum for biomedical engineering at higher education institutes is not updated and in line with the advancement of medical equipment, which is another challenge. The KIs mentioned that “Regular and specific training for the available medical equipment is neither provided to users nor to biomedical engineers working in the hospitals,” and “Most of the medical equipment failure is due to non-trained users of the medical equipment in most of the departments” (Informants #2, #4, #6, and #10). Another KI stated that “Even if some fragmented trainings are provided, it wasn't based on the hospital's medical equipment list, and redundant training didn't address the available medical equipment technology” (Informants #9 and #12).

Regarding the question about the maintenance and follow-up practices of the medical equipment at the surveyed facilities, some KIs reflected that the lack of availability of preventive maintenance for selected equipment was not their problem, saying that “even if the corrective maintenance is dependent on the type of the equipment, for our hospital, there is clearly scheduled preventive maintenance for emergency, ICU, and NICU medical equipment; otherwise, preventive maintenance is conducted as needed in other departments” (Informant #10).

“Planned preventive maintenance is mostly for emergency department equipment” (Informant # 6).

“If the defect is easy to maintain, the equipment is maintained by the hospital's biomedical engineering professionals after the work order is filled and reported by users” (Informant #11).

## Discussion

This study aimed to assess the availability and utilization of medical equipment and the major barriers that affect the utilization of medical equipment in comprehensive specialized hospitals in the Amhara Region, Ethiopia. Research on the usage of medical equipment contributes valuable insights for improvements in the health service delivery system at CSHs by minimizing the barriers to the access and utilization of capital medical equipment (4). Suboptimal access to diagnostic and therapeutic tools hampers the ability of healthcare providers to deliver timely and accurate care, potentially compromising patient outcomes and leading to low levels of patient satisfaction and preventable deaths (2, 10). This study revealed that the availability of at least one piece of capital medical equipment in the surveyed hospitals was only 25.6%, and the average availability of medical equipment in CSHs was only 55.93%, which indicates a notable deficiency in this fundamental aspect of healthcare infrastructure. Furthermore, the availability of critical medical equipment, such as machines for CT scans, MRI, x-ray radiography with digital fluoroscopy, and x-ray mammography, was inadequate across most of the hospitals surveyed for this study. The current result was somewhat consistent with the result of another study conducted in Ethiopia, it showed the presence of no-functional medical equipment at the health facilities (2). A noteworthy observation from our study was the substantial variability in the availability of medical equipment among the surveyed hospitals. The observed inadequacies in medical equipment availability have profound implications for healthcare delivery in comprehensive specialized hospitals. This finding is in line with the result of similar studies conducted in Jimma University Specialized Hospitals and Nekemte General Hospital, indicating a consistent trend of poor availability that varies across institutions (4). In addition, a study conducted in South Africa supported the result of the current study in that there was a lack of availability and inadequate maintenance of medical equipment in hospitals (2). Thus, the intended service could not be delivered in each hospital due to either the non-availability of medical equipment or a non-functionality problem. The inadequate access to medical equipment observed in the current study could be linked to the fact that, currently, in the region, many hospitals are upgrading from being general facilities to comprehensive specialized hospitals and there is a lack of awareness about the maintenance of medical equipment. Furthermore, the government may not be providing an adequate budget for the sector. Thus, the transition has seemingly occurred without the concurrent provision of essential medical equipment and other service requirements. Based on our observations from the survey, the biomedical engineers and pharmacy heads, crucial figures in the management of medical equipment, were found to be unfamiliar with the list of capital medical equipment. In addition to the quantitative findings, the qualitative results indicate that “regular and specific training about the available medical equipment is neither provided to users nor to biomedical engineers found in the hospitals. The majority of the hospitals don't have well-equipped functional workshops and well-trained personnel. Additionally, there is no national medical equipment maintenance workshop to serve as a training center. Even if some fragmented trainings were provided to biomedical professionals, the training is not based on the hospital's available medical equipment list and does not align with the current technologies” (Informant #2, #4, #6, #9, #10, and #12). The availability of equipment standards is an important factor when making an informed choice about what to buy to meet priority health needs and avoid wasting limited resources on devices that will not promote quality care, which is one of the main pillars of universal health coverage (14). A previous study also indicated that in Ethiopia, medical equipment development plans were not found in half of the surveyed public hospitals (15). However, according to this study, a lack of guidelines, which are important for complying with standards, could be a significant barrier to the acquisition of medical equipment in accordance with established guidelines. The qualitative findings of the present study also indicate “there is no any medical equipment decommission guideline in the hospitals and even in the ministry of health. There were no written documents to manage the hospital medical equipment” (Informant #4, #7, #9, #16).

The functionality and maintenance of medical equipment have a pivotal role in providing successful public healthcare services (16). If medical equipment is not functional and maintained, it has immediate impacts on healthcare delivery. Furthermore, the long-term storage of non-functional equipment incurs additional costs, including holding inventory costs, disposal expenses, and handling charges. This study also revealed a significant prevalence of non-functional medical equipment in the surveyed comprehensive specialized hospitals. According to this study, of the medical equipment available in the surveyed facilities, almost 26% was non-functional. The no-functionality status of medical equipment varied markedly from one hospital to another, with rates ranging from 43.9% in DBCSH to 13.9% in DCSH. However, the highest prevalence of functional medical equipment (86.6%) was found at DCSH, followed by DBCSH, where 57.3% of equipment was functional. The cumulative monetary value of all non-functional medical equipment was 368,349,102.42 Ethiopian Birr (ETB), suggesting a need for a more systematic approach to equipment management. This is somewhat similar to the result of an institutional-based cross-sectional study conducted in East Welega, Ethiopia, which reported notable rates of non-functional medical equipment (10). Different reasons have been indicated for the non-functionality of medical equipment. Therefore, the functionality and maintenance-associated challenges related to the use of medical equipment extend beyond the technical realm of equipment functionality; they directly impact the quality and reliability of healthcare services provided to patients.

The variation in the non-functionality status of medical equipment indicated by this study might be due to the commitment of some facilities to practice planned maintenance, as well as the access to spare parts, availability of skilled biomedical engineers, and implementation of MEMIS. The qualitative findings of this study indicated that in some surveyed facilities, the lack of availability of preventive maintenance for selected equipment was perceived to not be their problem, which was evidenced by the KIs: “even if the corrective maintenance is dependent on the type of the equipment, for our hospital, there is clearly scheduled preventive maintenance for emergency, ICU, and NICU medical equipment; otherwise, preventive maintenance is conducted as needed in other departments” (Informant #10).

The inability to maintain medical equipment not only jeopardizes diagnostic and treatment capabilities but also compromises patient safety and the overall efficiency of healthcare delivery (17). In the surveyed CSHs, several significant barriers emerged as primary contributors to the non-functionality of medical equipment. According to this study, the lack of spare parts and accessories accounted for 50.9% of the total, followed by the difficulty of equipment installation (17.3%). The result of this study is similar to the result of a study conducted in Ayder Hospital, Tigray, Ethiopia, which reinforces the notion that spare part scarcity is a prevalent issue affecting the maintenance of medical equipment (11). A similar study conducted in a tertiary setting revealed that the major critical barriers affecting the utilization of medical equipment were the non-availability of spare parts and maintenance (5, 18).

In addition to the quantitative findings, the qualitative results indicated that a lack of spare parts and accessories was the most frequent challenge, as mentioned by the KIs: “As you know, according to HSTG Chapter 15, donated medical equipment should be delivered with a two-year supply of accessories and spare parts, along with a one-year warranty period; however, most donated medical equipment is not provided according to donation guidelines, and without an operation and maintenance manual, it lacks spare parts and accessories. To be honest, most donor institutions considered Ethiopia a disposal site for medical equipment because a lot of old medical equipment was delivered through donations with incomplete accessories, rendering them unable to provide the intended service. In our hospital, there is much nonfunctional medical equipment, which is due to the absence of spare parts and accessories” (Informant #4, #7 and #16). Another KI also added that “the increased number of brands (e.g., Microscope has more than 20 brands) hinders the availability of specific accessories and spare parts. It is difficult to make purchases for all due to the limited number of suppliers and the non-availability of spare parts.” One KI emphasized that “in our hospital, we have a list of 800 pieces of medical equipment but are unable to obtain spare parts and accessories. The hospital can only procure spare parts for approximately 20 pieces of medical equipment per year” (Informant #3). Therefore, it is difficult to provide quality healthcare services.

Beyond spare part shortages, the qualitative study identified various barriers contributing to the non-functionality of medical equipment, including sources of medical equipment, the absence of a well-equipped a workshop and standard medical equipment installation and maintenance procedures, inadequate planned preventive maintenance, a lack of training for medical professionals on equipment usage, the closed-system nature of most laboratory equipment, the availability of several brands, a lack of training for the biomedical engineering department on how to maintain equipment, equipment not being installed, and a lack of budget for maintenance. Furthermore, professional-related, medical equipment-related, and health-facility-related aspects were the major issues affecting the availability, utilization, and functionality of medical equipment in the surveyed comprehensive specialized hospitals. Similarly, previous studies in Ethiopia also identified the multifaceted nature of factors contributing to the non-functionality of medical equipment (8, 19).

## Strengths and limitations of the study

Our study provides valuable insights into the state of medical equipment in CSHs in the Amhara Region. However, the scope of the current study was comprehensive specialized hospitals exclusively rather than assessing its availability and related status at other facilities such as primary hospitals and health centers. This targeted approach might limit the generalizability of our findings to other healthcare settings in the region. Therefore, for a more comprehensive understanding of the broader healthcare landscape, future studies should encompass a wider range of healthcare institutions and a more extensive inventory of medical equipment. Another limitation is the exclusion of health professionals directly involved in the daily use of medical equipment as the inclusion of these stakeholders would help capture a more nuanced picture of the barriers and facilitators to the use of medical equipment in the healthcare delivery system. Given the aforementioned limitations, future research endeavors should aim to address these gaps for a more holistic understanding of the availability and functionality of medical equipment.

## Conclusion and recommendation

In this study, the availability, functionality, and barriers related to the use of medical equipment at eight CSHs in the Amhara Region, Ethiopia, were assessed, and the study revealed the presence of critical deficiencies in the availability and functionality of medical equipment in CSHs in the Amhara Region, Ethiopia. Challenges include a shortage of spare parts and accessories, the absence of a well-equipped and standardized maintenance workshop, training gaps, maintenance and installation issues, and budget constraints. Addressing these challenges requires a collaborative effort, strategic investments, and future research to broaden the scope and consider a wider range of healthcare settings. Therefore, concerned bodies such as the CSHs, ARHB, EPSS, and MOH should work cooperatively and exert maximal effort to improve the availability and accessibility of medical equipment through the allocation of an adequate budget, implement regulations and policies on medical equipment utilization and management practices, provide regular capacity building and training programs for biomedical technicians, and the improve equipment maintenance facilities at each health service delivery point.

## Data Availability

The raw data supporting the conclusions of this article will be made available by the authors, without undue reservation.
