# Supplementary material for: The availability and functionality of medical equipment and the barriers to their use at comprehensive specialized hospitals in the Amhara region, Ethiopia
Source: Front Health Serv. 2025 Jan 7;4:1470234. doi: 10.3389/frhs.2024.1470234 (PMC11748297; doi:10.3389/frhs.2024.1470234)
Supplement: Supplementary file 1 [file Table1.docx]

Supplementary table 1: Key informant interview guideline

| s.no | Open Ended | Ways of Probe |
| --- | --- | --- |
| 1 | How do you obtain medical equipment? | Is by donation (if open/closed) or purchasing? |
| 2 | How do you install and maintain medical equipment | Who is responsible for installation and/or maintenance of medical equipment? Is the Hospital or the manufacturer or the agent responsible? Is there the necessary tool kit in the hospital? Is medical equipment installed as the time of delivery If not why?  Is there user training provided during installation or after installation by Biomedical or  Not if not why? If yes how? |
| 3 | Do you think the hospital is managing medical equipment appropriately? What is the hospital strategy used to manage medical equipment? | How to manage, is there written medical equipment strategic management document, is there user training plan, was training provided to biomedical regularly? If yes when? By whom? If no, why, and how to use it? Main reasons for Non-functionality, how do you monitor the management of medical equipment? |
| 4 | What are the critical barriers/challenges in your hospital to utilize the medical equipment efficiently/ reasons for non- functionality and your recommendation  for improvement | Is their regular training for professionals, how do you see the power supply to the hospitals ,  Is their sufficient accessory and spare part  Is there any other challenges specific to your HL? what will be the solution and what you suggest  To solve the problem? |

Thank you!
