# Supplementary material for: The availability and functionality of medical equipment and the barriers to their use at comprehensive specialized hospitals in the Amhara region, Ethiopia
Source: Front Health Serv. 2025 Jan 7;4:1470234. doi: 10.3389/frhs.2024.1470234 (PMC11748297; doi:10.3389/frhs.2024.1470234)
Supplement: Supplementary file 2 [file Table2.docx]

Supplementary table 2: Professional related factors on the overall aspects of medical equipment utilization and related issues

| **Related items Response** | | |
| --- | --- | --- |
|  | | |
| **Professional related factors** Yes | | No |
| Does the hospital have a biomedical engineer for the management of medical equipment? | 8 | 0 |
| Does the biomedical engineer in the hospital trained in how to maintain and repair different medical equipment? | 4 | 4 |
| Does the Biomedical engineer obtain refresher training regularly when the arrival of new medical equipment | 2 | 6 |
| Do Staffs who works with equipment/operator are usually trained well in advance for proper use of ME. | 5 | 3 |
| Does the biomedical engineer involve in the purchase or donation medical equipment before final decision | 5 | 3 |
| Does the Biomedical engineer always maintain medical equipment when difficulty of operation? | 7 | 1 |
