# Supplementary material for: The availability and functionality of medical equipment and the barriers to their use at comprehensive specialized hospitals in the Amhara region, Ethiopia
Source: Front Health Serv. 2025 Jan 7;4:1470234. doi: 10.3389/frhs.2024.1470234 (PMC11748297; doi:10.3389/frhs.2024.1470234)
Supplement: Supplementary file 3 [file Table3.docx]

Supplementary table 3: Medical equipment related factors on the overall aspects of medical equipment utilization and related issues

| **Related items** | **Response** | |
| --- | --- | --- |
| **Medical equipment related** | Yes | No |
| Do all medical equipment installed as soon as delivered to the hospital. | 2 | 6 |
| Does the hospital have preventive maintenance schedule for medical equipment? | 5 | 3 |
| Do the hospital avail spare parts before medical equipment stopped working? | 1 | 7 |
| Does the medical equipment have an accessory as the nature of the equipment? | 1 | 7 |
| Do medical equipment in the hospital stored in good condition? | 4 | 4 |
| Does Medical Equipment preventive maintenance practical base on demand of the equipment in the schedule | 2 | 6 |
