# Supplementary material for: The availability and functionality of medical equipment and the barriers to their use at comprehensive specialized hospitals in the Amhara region, Ethiopia
Source: Front Health Serv. 2025 Jan 7;4:1470234. doi: 10.3389/frhs.2024.1470234 (PMC11748297; doi:10.3389/frhs.2024.1470234)
Supplement: Supplementary file 4 [file Table4.docx]

Supplementary table 4: Health facility related factors on the overall aspects of medical equipment utilization and related issues

| **Related items** | | | Response | | | |
| --- | --- | --- | --- | --- | --- | --- |
| **Health facility related** | | | Yes | No | | |
| Does the hospital have a detailed medical equipment policy that includes preventive maintenance and for use? | | | 6 | 2 | | |
| Does the medical equipment policy effectively and adequately used? | | | 1 | 7 | | |
| Does the Hospital have medical equipment operating manual? | | | 4 | 4 | | |
| Does the hospital have medical equipment preventive maintenance schedule based on the nature of the equipment? | | | 6 | 2 | | |
| Does the Hospital have medical equipment maintenance workshop? | | | 6 | 2 | | |
| All stakeholders are usually involved before equipment is purchased/requisition | | | 5 | 3 | | |
| The hospital continuously monitors and evaluates the use of the medial equipment | | | 3 | 5 | | |
| The staffs are well informed of the medical equipment policy at the hospital | | | 1 | 7 | | |
| The department did a report for the uninstalled, non-functional, and spare part shortage of medical equipment regularly to decision maker? | | | 8 | 0 | | |
| Preventive maintenance ensures that the equipment is safe for the patients and staff | | | 8 | 0 | | |
| Do the medical equipment storage space enough for the available medical equipment and appropriately stored in a good storage condition? | 1 | | | 7 |  |  |
| When medical equipment breaks down, they are repaired immediately | 7 | | | 1 |  |  |
| Is the medical equipment served only in its intended department and purpose only? | 8 | | | 0 |  |  |
| Does the hospital have enough power to operate all medical equipment? | 4 | | | 4 |  |  |
